# ALK-positive anaplastic large cell lymphoma in adults

**DOI:** 10.12703/r/12-21

**Published:** 2023-08-25

**Authors:** Matthew J Gromowsky, Christopher R D’Angelo, Matthew A Lunning, James O Armitage

**Affiliations:** 1 University of Nebraska Medical Center, Omaha, Nebraska, USA

**Keywords:** Lymphoma, ALCL, PTCL, ALK positive ALCL

## Abstract

ALK-positive anaplastic large cell lymphoma (ALCL) represents approximately 6–7% of the mature T-cell lymphomas. This subtype contains a translocation between the ALK gene on chromosome 2 and one of several other genes that together form an oncogene. The most frequent translocation is t(2;5) which combines ALK with NPM1. This lymphoma has a median age of 34 years, is more common in males, and is in advanced stage at the time of diagnosis in most patients. ALK-positive ALCL is the most curable of the peripheral T-cell lymphomas. The CHOP regimen has been most frequently used, but results are improved with the substitution of brentuximab vedotin for vincristine (BV-CHP) and the addition of etoposide (CHOEP), with BV-CHP being favored. Salvage therapies include allogeneic or autologous bone marrow transplantation, BV, if not used as part of the primary therapy, and ALK inhibitors. The latter are very active and likely to be incorporated into the primary therapy.

## Introduction

Anaplastic large cell lymphoma (ALCL) is a type of mature peripheral T-cell lymphoma (PTCL) that makes up about 2% of non-Hodgkin’s lymphoma diagnoses^1^. There are multiple types of ALCL, including primary cutaneous ALCL (PC ALCL), systemic ALCL, and breast-implant-associated ALCL, each of which shares the pathologic characteristics of sinusoidal invasion and a CD30+ immunophenotype^2^. Of these, only systemic ALCL commonly expresses the anaplastic lymphoma kinase (ALK) gene. This subtype, ALK+ ALCL, contains a translocation between the ALK gene that is located in chromosome region 2p23.2-p23.1 and is one of several genes that together form an oncogene, the most frequent translocation being a t(2;5) translocation that combines ALK with NPM1^3^.

ALK+ ALCL is rare in adults (people over the age of 18), and it is especially rare in people over the age of 50, yet it is one of the most common pediatric lymphomas after Burkitt lymphoma, Burkitt-like lymphoma, lymphoblastic T-cell lymphomas, and diffuse large B-cell lymphoma. Compared to other sub-types of PTCL, ALK+ ALCL bears a significantly better prognosis, with front-line treatment often being curative. Unfortunately, relapsed and refractory disease bears a worse prognosis, and there is no standard on how to treat relapsed or refractory disease. The purpose of this review is to discuss the features of ALK+ ALCL to gain a better understanding of the disease, to define the current challenges and frontiers in the management of ALK+ ALCL, and to review current and future strategies to mitigate these challenges.

## Epidemiology

It is estimated that approximately 96–160 adult patients are diagnosed with ALK+ ALCL in the United States each year^1,4^. The ALK+ subtype of ALCL has a male-to-female ratio of 1.5 and is commonly found in a younger population than the other ALCL subtypes. The median age of onset is 34 years old and can present at ages as young as pre-teen years^5^. ALCL makes up between 10 and 15 percent of lymphoma diagnoses in children. In adults, the majority of systemic ALCL is ALK-, with about 50–60% of adult ALCL cases being ALK-^6^.

In an analysis of the adult population of patients with ALK+ ALCL, it was found that the disease had a higher incidence in Blacks than in other demographics, with American Indians and Asian/Pacific Islanders having the least incidence^7^. Certain racial and socioeconomic disparities exist in treatment outcomes for patients with ALK+ ALCL. Caucasian patients were found to have better outcomes than African Americans^7^.

## Pathophysiology: ALK

ALK, part of the primary driver mutation in ALK+ ALCL, was first discovered in 1994 in ALCL cell lines that contained the NPM-ALK fusion^3^. The ALK gene is located on chromosome 2, is 1620 amino acids long, and plays a role in several malignancies besides ALCL, including neuroblastomas and NSCLC. PC-ALCL may also have ALK expression, but this is much less common than the ALK-PC-ALCL variety^8^. ALK encodes for a receptor tyrosine kinase (RTK), catalyzing the transfer of the gamma phosphate group of adenosine triphosphate (ATP) to a tyrosine residue on its substrate protein, which can help activate downstream pathways^3^. ALK contains an extracellular domain that includes a low-density lipoprotein receptor class A domain, a hydrophobic, single-pass transmembrane domain, and an intracellular kinase domain^3^. The intracellular domain contains several loop regions and beta-strands with a single αC-helix, and activation is terminated through dephosphorylation of the tyrosine phosphatase, endocytosis, and degradation^3^.

ALK is expressed in adult mammals in the hippocampus, dentate gyrus, and Cornu ammonis, with its expression greatest before birth^3^. Little is known about the function of ALK, but it may affect behavior and the development of the brain and motor neurons^3^. ALK has been shown to activate several signaling pathways, including RAS/MAPK, JAK/STAT, and PI3K/Akt^3^. The downstream RAS/MAPK and JAK/STAT pathways, which play a role in cell growth and division, may be some of the largest drivers of the oncogenic effects of ALK^9^.

Translocation of ALK to nucleophosmin 1 (NPM1) is the most common driver mutation of ALK+ ALCL, but other translocations can occur. The NPM-ALK fusion causes aberrant ALK gene expression that activates a variety of signaling pathways that can cause uncontrolled cellular proliferation. NPM1 is located on chromosome 5 and encodes for a chaperone protein that is generally located in the nucleus^10^. NPM1 participates in cell cycle progression, apoptosis, and ribosomal biogenesis, among other functions^10^. Mutations in NPM1 are the most frequent genetic abnormality found in AML^10^. Fusion of ALK with NPM1 encodes a chimeric protein that contains the amino terminus of NPM1 and the catalytic domain of ALK^11,12^. This leads to ALK being constitutively active, thus activating many of its downstream pathways.

## Diagnosis (see Figure 1)

By definition, ALK+ ALCL must express CD30 as well as ALK. Among expert hematologists, there is a diagnostic concordance rate of about 82.3% when diagnosing ALK+ ALCL^13^. CD30 expression is not unique to ALCL and is expressed in normal activated B and T cells, as well as other malignancies such as classical Hodgkin lymphoma and other B-cell lymphomas^14^. On pathologic evaluation, ALCL often contains characteristic cellular architecture that includes an eccentric, horseshoe-shaped nucleus with an eosinophilic region near the nucleus that is found in 60% of cases^15,16^. Other cellular patterns exist that include a small cell pattern showing small-to-medium-sized neoplastic cells with irregular nuclei, a composite form, a lymphohistiocytic form, and a Hodgkin-like pattern^15,17^. Of these patterns, the small cell and lymphohistiocytic forms may be associated with a worse prognosis^18^. In as many as 15% of cases, more than one pattern is observed^15^.

**Figure 1.**
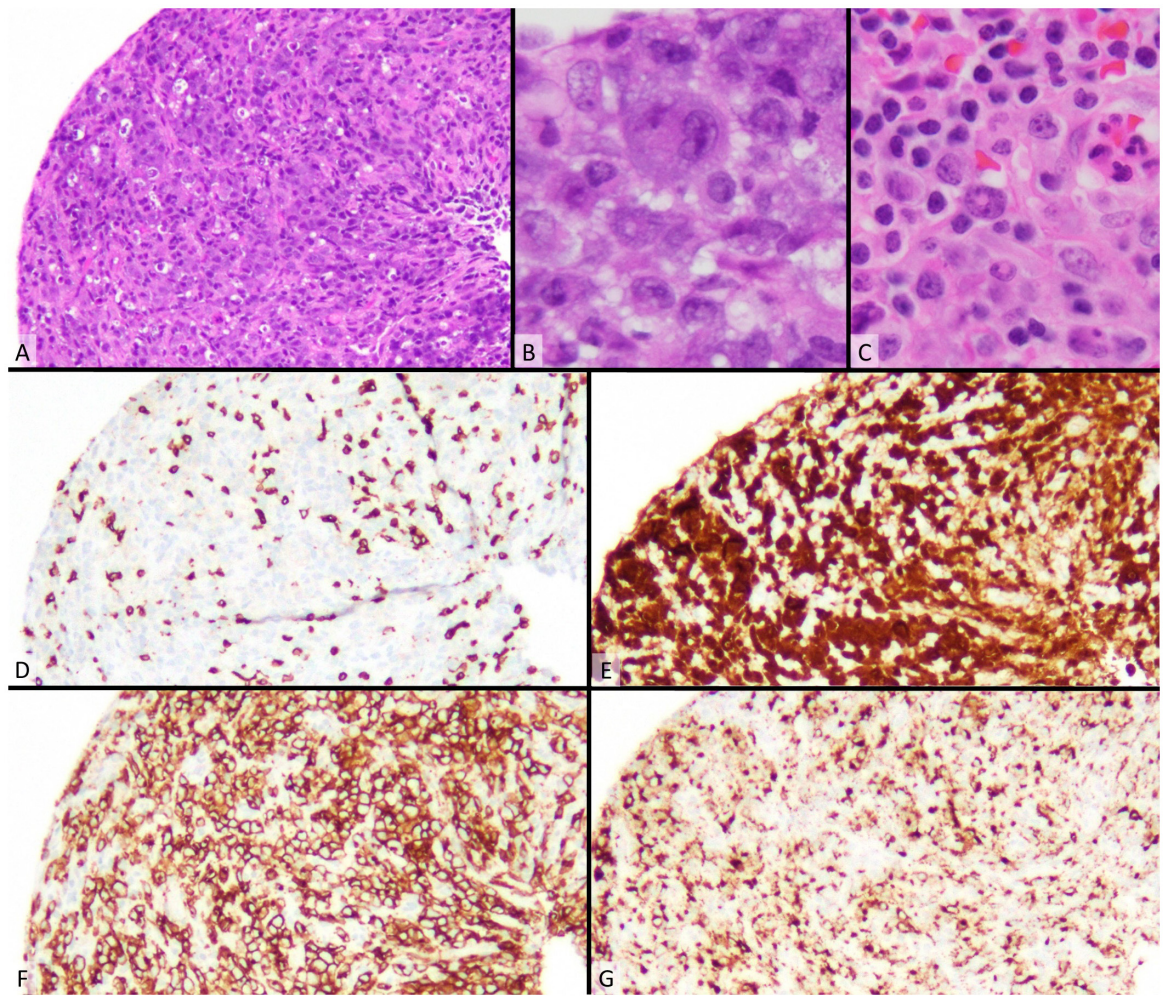
Morphology and Immunohistochemistry of ALCL. **A**) By H&E (100x), the common pattern of ALCL shows pleomorphic cells. **B**) At higher power (400x), there are hallmark cells with reniform nuclei and variably eosinophilic, perinuclear cytoplasm. **C**) H&E (400x) The small cell variant of ALCL has a predominance of small cells with irregular nuclei and fewer hallmark cells. **D**–**G**) The immunostains correspond to the common pattern in panel **A**. The CD3 (D, 100x) is negative in the neoplastic cells, while ALK (E, 100x), CD30 (F, 100x), and granzyme B (G, 100x) are positive.

Immunohistochemistry should be performed for diagnosis. Stains that support an ALK+ ALCL diagnosis include CD30, ALK, EMA, CD2, CD4, CD5, TIA1, granzyme B, perforin, CD45, CD61, CD25, and BNH9^19^. Importantly, T-cell markers including CD3 may also be negative^18^. In ALK+ ALCL, CD30 is often present in the Golgi and cell membrane region, while ALK is located in the cytoplasmic and nuclear region^15^. ALK expression is nearly synonymous with ALK rearrangement^20^. Therefore, ALK staining is usually used to distinguish between ALK+ and ALK- ALCL; they are identical besides ALK expression and the fact that ALK- ALCL lacks the small cell pattern^20^. Finally, the pattern and location of ALK staining may relate to the fusion partner of ALK, where ALK-NPM fusion involves some degree of nuclear staining, and ALK detection in the cytoplasm alone may relate to an alternative fusion partner^18^.

## Presentation and Evaluation (see Table 1)

ALK+ ALCL often presents with systemic symptoms that can include weight loss, fever, weakness, fatigue, and night sweats with concurrent lymphadenopathy^15^. The disease is usually advanced upon presentation, with approximately 65% of cases presenting in stage III or IV^15^.

**Table 1.** A comparison of several PTCLs^5,27^.

Aggressive PTCL Subtypes frequent in North America and Europe	Percent of all PTCL/ NK cell lymphomas diagnosed/year	Percent Stage III or IV at diagnosis	Median Age	Percent Male	Percent Bone Marrow Positivity	Percent CD-30 positivity**
PTCL NOS*	25.9	67	60	66	22	64.36 (56/87)
ALK+ ALCL*	6.6	65	34	63	12	100
ALK- ALCL*	5.5	71	58	61	7	100
AITL*	18.5	89	65	56	29	42.84 (18/42)
Enteropathy-Associated PTCL*	4.7	69	61	53	3	75 (9/12)
**Hepatosplenic Lymphoma**	1.4	95	34	68	74	Not Available
SPTCL*	0.9	83	33	75	6	Not Available

*PTCL NOS - Peripheral T-Cell Lymphoma Not Otherwise Specified, ALK+ ALCL - Anaplastic Lymphoma Kinase-Positive Anaplastic Large Cell Lymphoma, ALK- ALCL - Anaplastic Lymphoma Kinase-Negative Anaplastic Large Cell Lymphoma, AITL – Angioimmunoblastic T-Cell Lymphoma, Enteropathy-Associated PTCL – Enteropathy-Associated Peripheral T-Cell Lymphoma, SPTCL – Subcutaneous Panniculitis Like T-Cell Lymphoma. **Tumors considered CD30 Positive if greater than zero CD30 positive cells in the sample.

After histological diagnosis, staging should include bloodwork, imaging (preferably PET/CT), and a bone marrow biopsy. Adverse prognostic factors include advanced stage, advanced age, and bone marrow involvement^15^. Rarely, ALCL can present in the CNS, which correlates with a worse prognosis^21,22^.

## Primary Therapy (see Table 2)

Unlike many T-cell lymphomas, ALK+ ALCL can often be cured with the cyclophosphamide, doxorubicin, vincristine, and prednisone (CHOP) regimen. Therefore, the primary therapy for ALK+ ALCL has generally consisted of CHOP or CHOP-like regimens^23^.

**Table 2.** A comparison between ALK+ and ALK- ALCL treatments and outcomes^5,20,23,28^.

Subtype	Front-line therapy	CR rate	5-year PFS	Median Survival	5-year survival
**ALK+**	(BV)-CHP, CHOP, CHOEP	85%	69%	>10 years	70–90%
**ALK-**	(BV)-CHP, CHOP, CHOEP	63%	43%	4.5 years	40–60%

Recently, brentuximab vedotin (BV) has been integrated into front-line therapies for ALK+ ALCL. Brentuximab vedotin is a chimeric monoclonal antibody-drug conjugate that targets CD30, the surface antigen universally expressed in ALCL but also expressed in other lymphomas, including Hodgkin lymphoma and some DLBCLs^24,25^. Upon binding to the antigen, the drug is internalized, and the monomethyl auristatin E (MMAE) payload that is coupled to the antibody is released to bind to tubulin and cause cell cycle arrest^26^. While primary therapies may change as newer drugs are approved, BV remains part of the current standard of care for ALK+ ALCL as a part of the BV-CHP regimen (CHOP with BV substituted for vincristine).

The ECHELON-2 trial was a phase III, global trial comparing cyclophosphamide, doxorubicin, vincristine, and prednisone (CHOP) to cyclophosphamide, doxorubicin, prednisone, and brentuximab vedotin (BV-CHP) when treating CD30+ lymphomas^29^. BV-CHP was superior to CHOP based on significant improvement in progression-free survival and overall survival with similar safety profiles^29^. The similar safety profile of BV-CHP to CHOP is unique because many other CHOP-like regimens that have been used show much greater toxicities^29^. The study found a three-year OS of 76.8% and 69.1% for BV-CHP and CHOP, respectively, and a three-year PFS of 57.1% and 44.4% for BV-CHP and CHOP^29^. We consider BV-CHP to be the new standard of care based on the results of ECHELON-2.

If BV is not able to be given for various reasons, including lack of access, cost, or pre-existing neuropathy, we recommend giving CHOP or a CHOP-like regimen. There is some indication that the addition of etoposide to the CHOP regimen (CHOEP) could lead to a greater survival benefit than CHOP alone, especially in patients with a normal LDH^30^. Studies have compared these two regimens, including one that showed an 86% vs 61% CR and a 90% vs 61% 5-year survival for CHOEP vs CHOP, respectively, in patients with ALK+ ALCL^31^. Another study found a 3-year OS advantage of 92% to 49% for CHOEP compared to CHOP, but they had a limited patient population^23^. A recent meta-analysis found no difference between CHOP and CHOEP when treating mixed PTCLs, but other studies have shown a favorable response to CHOEP over CHOP for treating ALK+ ALCL^23,30,32^. For patients unable to receive BV, we would generally recommend CHOEP in younger patients or CHOP for patients unable to tolerate etoposide.

While standard treatments for PTCL include consolidative stem cell transplantation, this is not usually recommended for ALK+ ALCL because of the durability of remissions seen after completion of first-line treatments^33^. For example, the ECHELON-2 trial did not mandate a transplant after front-line therapy, and patients with ALK+ ALCL fared remarkably well compared to other PTCL subtypes^29^.

## Salvage therapy

### Relapsed/Refractory Disease

While primary therapy for ALK+ ALCL is well-defined, there is no consensus on the treatment for relapsed or refractory disease. Currently, stem cell transplantation, platinum-based regimens, and ALK inhibitors are some of the most used options for the treatment of relapsed or refractory disease. It should be noted that patients with disease relapse generally have a better outlook than those with refractory disease, but the treatment options remain similar.

For refractory disease, we favor treating with chemotherapy regimens that have alternative mechanisms of action to CHOP or BV-CHP. An example of an appropriate and commonly used therapy would be ICE, which contains ifosfamide, carboplatin, and etoposide^34^.

Some other drugs that should be considered and are FDA approved for relapsed disease include belinostat, pralatrexate, and romidepsin. An emerging option may be ruxolitinib, a JAK1/2 inhibitor that has been shown to be effective in treating relapsed or refractory PTCLs, especially those with JAK/STAT active signaling given to downstream activity of ALK^35^.

Autologous hematopoietic stem cell transplantation (auto-HCT) following high-dose chemotherapy remains an option for the treatment of relapsed ALK+ ALCL. Allogeneic stem cell transplantation (Allo-HCT) may also play a role in the treatment of refractory disease, although it is associated with a much higher rate of treatment mortality. There remains debate over whether auto-HCT or allo-HCT should be pursued in patients with 1^st^ relapsed or refractory ALCL. A large cohort study performed by the Center for International Blood and Marrow Transplant Research (CIBMTR) found that patients with relapsed ALCL undergoing auto-HCT had superior outcomes to those receiving allo-HCT with a smaller nonrelapse mortality at 100 days, 1 year, and 3 years, and superior PFS and OS at 1 and 3 years for the auto-HCT patients compared to allo-HCT patients, although this study also did not separate ALCL based on ALK status and patients had varying baseline characteristics^36^. An analysis of the CIBMTR database of 182 ALCL patients receiving allo-HCT that similarly did not separate patients based on ALK status did find that allo-HCT for relapsed/refractory ALCL had a 5-year pFS of 41% and OS of 56%, indicating that allo-HCT can play an effective role in treating relapsed/refractory disease^37^. A recent meta-analysis comparing auto-HCT to allo-HCT in relapsed/refractory PTCL patients showed benefits to both types of HCT depending on the patient population, finding some benefit to allo-HCT in patients with a higher-risk disease stage agent^38^.

Based on the demonstrated efficacy for allogeneic transplant, we favor proceeding with an allogeneic transplant, especially for patients who are refractory to BV-containing-induction regimen, fit, and possess few comorbidities. We reserve autologous transplant for patients with relapsed disease or those without a suitable donor.

The future of treating all forms of systemic ALCL may include anti-CD-30 chimeric antigen receptor T-cells (CAR-T) cells. So far, there has been limited success in treating ALCL in this manner, but third-generation anti-CD30 CAR-T cells may show promise^39^.

ALK inhibitors are a more recently approved therapy that are often given as a stand-alone therapy in relapsed or refractory disease. Crizotinib, a tyrosine kinase ALK inhibitor, was approved for treating refractory ALK+ ALCL in pediatric patients and young adults in January of 2021^40^. To this date, the ideal duration of therapy has not been determined, but it has been given for up to 37 months or more^41^. Initially, crizotinib was developed for the treatment of ALK+ NSCLC in adults, but it has since entered ALCL young adult and pediatric treatment regimens^6^. Use of crizotinib in older adults with relapsed or refractory ALK+ ALCL has not been approved, but a trial for adults is currently underway^6,40^. A small single-center study that evaluated crizotinib as a monotherapy for patients with relapsed or refractory ALK+ ALCL, ALK+ DLBCL, and ALK+ PBL with 25, 1, and 1 patients, respectively, found a durable remission in almost 2/3 of the patients^42^. Trials are also being explored to use an ALK inhibitor as a part of front-line therapy for ALK+ ALCL, but to date, it is still only approved for relapsed or refractory disease^6^.

Ceritinib is another ALK inhibitor that has shown promise in the treatment of both ALK+ NSCLC and ALK+ ALCL, but its use may be limited based on toxicities^6^. However, it has shown long-lasting responses in a small number of adults with ALK+ ALCL in the Phase I ASCEND-1 trial (registered as NCT01283516), which included 3 patients with relapsed ALK+ ALCL^6,43^.

Alectinib, a second-generation oral ALK inhibitor, has been shown to have a superior effect in treating ALK+ NSCLC compared to crizotinib in a large randomized phase III study^44^. Additionally, alectinib is able to cross the blood-brain barrier, indicating that it may be an effective option for patients with CNS involvement by ALK+ ALCL. A single-arm, phase II study was published in 2020 that tested the efficacy of alectinib in ten patients with relapsed or refractory ALK+ ALCL^45^. Of these patients, eight (80%) attained an objective response, with six patients (60%) attaining a CR^45^. Because of these favorable results, it is likely that alectinib will play a role in ALK+ ALCL treatment in the future.

## Pediatric Advances

While the main focus of this review is ALCL in adults, advances in pediatric ALK+ ALCL must be acknowledged. Similar to therapy in adults, ALK-inhibitors such as crizotinib and brentuximab vedotin are improving outcomes in children and generally adding few toxicities to treatment^46–48^. In children, it has been found that minimum disseminated disease (MDD) and minimum residual disease (MRD) have been useful prognostic factors for determining whether patients are at high risk for relapse^49–51^. To determine whether there is MDD or MRD, reverse-transcriptase polymerase chain reaction (RT-PCR) of bone marrow or blood samples was run, and normalized copy numbers of NPM-ALK were determined^49,50^. In patients with more than 10 normalized copy numbers of NPM-ALK on quantitative RQ-PCR, there was a relapse rate of 61% compared to just 21% for those with fewer than 10 normalized copy numbers^49^. After treatment, MRD could be determined in a similar method using RT-PCR. In MDD+/MRD+ patients, 81% of patients relapsed as opposed to 31% of patients who were MDD+/MRD-^51^. Another detectable biological factor, the detection of >1/750 titers of ALK autoantibodies, correlated with a lower incidence of relapse^52,53^. MDD, MRD, and ALK autoantibodies can therefore be used to determine which patients may need to be more closely observed because of this correlation with relapse. It is likely that these biological factors could impact adult treatment as well, with MRD already being shown to correlate with PFS in adults^54^.

## Prognosis-Favorable Until it Isn’t

ALK+ ALCL is the most treatable form of systemic ALCL, with an overall response rate of up to 90%, a relapse-free survival of about 60%, and a 5-year overall survival of about 70%^15^. Compared to ALK- ALCL, these outcomes are far superior^5^. Part of this might come from the fact that ALK+ ALCL is seen in a younger population than ALK- disease^23^. In fact, several large studies have found that age >40 exerts a strong negative prognostic impact and is a better prognostic value than ALK expression in ALCL in a multivariate analysis^55^. However, treatment-refractory ALK+ ALCL has a less positive outlook, with a median PFS and OS of 3.8 and 13.6 months, respectively, for patients with first-relapsed or refractory disease according to the LYSA study, which was before brentuximab vedotin^56^. For patients who had not already been treated with brentuximab vedotin, single-agent brentuximab vedotin showed improved outcomes for patients with relapsed or refractory disease in a phase 2 clinical trial showing a median PFS of 25.5 months and a five-year OS of 56%^57^. The ECHELON-2 trial did find a 59% overall response rate to brentuximab vedotin retreatment after original treatment with BV-CHP, indicating that single-agent brentuximab vedotin is still an option for patients even with BV-CHP as the standard of care^29^.

## Future Directions/Conclusions

The ECHELON-2 trial has established BV-CHP as a new standard of front-line therapy for patients with ALK+ ALCL. In situations where BV-CHP cannot be given or is contraindicated, CHOP and CHOEP remain alternatives. For relapsed or refractory disease, treating patients with an alternative chemotherapy regimen followed by an autologous stem cell transplantation if chemosensitivity is demonstrated is a reasonable approach. For patients with primary refractory disease, we consider consolidative allogeneic transplantation if a remission can be achieved by an alternative chemotherapy regimen. We are encouraged by the initial success of ALK inhibitors as salvage therapy, and it is possible that they may eventually become a part of primary therapy.

## References

[1] A clinical evaluation of the International Lymphoma Study Group classification of non-Hodgkin's lymphoma. The Non-Hodgkin's Lymphoma Classification Project. *Blood.* 1997; 89(11): 3909–3918. 10.1182/blood.V89.11.3909 9166827

[2] Jaffe ES : Anaplastic large cell lymphoma: the shifting sands of diagnostic hematopathology. *Mod Pathol.* 2001; 14(3): 219–28. 10.1038/modpathol.3880289 11266530

[3] Huang H : Anaplastic Lymphoma Kinase (ALK) Receptor Tyrosine Kinase: A Catalytic Receptor with Many Faces. *Int J Mol Sci.* 2018; 19(11): 3448. 10.3390/ijms19113448 30400214 PMC6274813

[4] Marchi E O'Connor OA : The rapidly changing landscape in mature T-cell lymphoma (MTCL) biology and management. *CA Cancer J Clin.* 2020; 70(1): 47–70. 10.3322/caac.21589 31815293

[5] Vose J Armitage J Weisenburger D : International peripheral T-cell and natural killer/T-cell lymphoma study: pathology findings and clinical outcomes. *J Clin Oncol.* 2008; 26(25): 4124–30. 10.1200/JCO.2008.16.4558 18626005

[6] Prokoph N Larose H Lim MS : Treatment Options for Paediatric Anaplastic Large Cell Lymphoma (ALCL): Current Standard and beyond. *Cancers (Basel).* 2018; 10(4): 99. 10.3390/cancers10040099 29601554 PMC5923354

[7] Guru Murthy GS Hamadani M Bhatt VR : Systemic Anaplastic Lymphoma Kinase-positive Anaplastic Large Cell Lymphoma: A Population-based Analysis of Incidence and Survival. *Clin Lymphoma Myeloma Leuk.* 2017; 17(4): 201–206. 10.1016/j.clml.2017.02.003 28395812

[8] Liu HL Hoppe RT Kohler S : CD30^+^ cutaneous lymphoproliferative disorders: the Stanford experience in lymphomatoid papulosis and primary cutaneous anaplastic large cell lymphoma. *J Am Acad Dermatol.* 2003; 49(6): 1049–58. 10.1016/s0190-9622(03)02484-8 14639383

[9] Molina JR Adjei AA : The Ras/Raf/MAPK Pathway. * J Thorac Oncol.* 2006; 1(1): 7–9. 10.1016/S1556-0864(15)31506-9 17409820

[10] Rau R Brown P : Nucleophosmin (*NPM1*) mutations in adult and childhood acute myeloid leukaemia: towards definition of a new leukaemia entity. *Hematol Oncol.* 2009; 27(4): 171–81. 10.1002/hon.904 19569254 PMC3069851

[11] Gascoyne RD Aoun P Wu D : Prognostic significance of anaplastic lymphoma kinase (ALK) protein expression in adults with anaplastic large cell lymphoma. *Blood.* 1999; 93(11): 3913–21. 10.1182/blood.V93.11.3913 10339500

[12] Suzuki R Kagami Y Takeuchi K : Prognostic significance of CD56 expression for ALK-positive and ALK-negative anaplastic large-cell lymphoma of T/null cell phenotype. *Blood.* 2000; 96(9): 2993–3000. 11049976

[13] Laurent C Baron M Amara N : Impact of Expert Pathologic Review of Lymphoma Diagnosis: Study of Patients From the French Lymphopath Network. *J Clin Oncol.* 2017; 35(18): 2008–2017. 10.1200/JCO.2016.71.2083 28459613

[14] Montes-Mojarro IA Steinhilber J Bonzheim I : The Pathological Spectrum of Systemic Anaplastic Large Cell Lymphoma (ALCL). *Cancers (Basel).* 2018; 10(4): 107. 10.3390/cancers10040107 29617304 PMC5923362

[15] Ferreri AJM Govi S Pileri SA : Anaplastic large cell lymphoma, ALK-positive. *Crit Rev Oncol Hematol.* 2012; 83(2): 293–302. 10.1016/j.critrevonc.2012.02.005 22440390

[16] Benharroch D Meguerian-Bedoyan Z Lamant L : ALK-positive lymphoma: a single disease with a broad spectrum of morphology. *Blood.* 1998; 91(6): 2076–84. 10.1182/blood.V91.6.2076 9490693

[17] Vassallo J Lamant L Brugieres L : ALK-positive anaplastic large cell lymphoma mimicking nodular sclerosis Hodgkin's lymphoma: report of 10 cases. *Am J Surg Pathol.* 2006; 30(2): 223–9. 10.1097/01.pas.0000179123.66748.c2 16434897

[18] Lamant L McCarthy K d'Amore E : Prognostic impact of morphologic and phenotypic features of childhood ALK-positive anaplastic large-cell lymphoma: results of the ALCL99 study. *J Clin Oncol.* 2011; 29(35): 4669–76. 10.1200/JCO.2011.36.5411 22084369

[19] Kaseb H Mukkamalla SKR Rajasurya V : Anaplastic Large Cell Lymphoma. In: *StatPearls.* StatPearls Publishing, 2022. 30725835

[20] Hapgood G Savage KJ : The biology and management of systemic anaplastic large cell lymphoma. *Blood.* 2015; 126(1): 17–25. 10.1182/blood-2014-10-567461 25869285

[21] Dong X Li J Huo N : Primary central nervous system ALK-positive anaplastic large cell lymphoma in an adult: A rare case report. *Medicine (Baltimore).* 2016; 95(49): e5534. 10.1097/MD.0000000000005534 27930548 PMC5266020

[22] Ahrendsen JT Ta R Li J : Primary Central Nervous System Anaplastic Large Cell Lymphoma, ALK Positive. *Am J Clin Pathol.* 2022; 158(2): 300–310. 10.1093/ajcp/aqac046 35460414

[23] Sibon D Nguyen DP Schmitz N : ALK-positive anaplastic large-cell lymphoma in adults: an individual patient data pooled analysis of 263 patients. *Haematologica.* 2019; 104(12): e562–e565. 10.3324/haematol.2018.213512 31004022 PMC6959172

[24] Sharman JP Goldschmidt JH Burke JM : CD30 expression in nonlymphomatous malignancies. *J Clin Oncol.* 2012; 30(15_suppl): 3069–3069. 10.1200/jco.2012.30.15_suppl.3069

[25] Hu S Xu-Monette ZY Balasubramanyam A : CD30 expression defines a novel subgroup of diffuse large B-cell lymphoma with favorable prognosis and distinct gene expression signature: a report from the International DLBCL Rituximab-CHOP Consortium Program Study. *Blood.* 2013; 121(14): 2715–24. 10.1182/blood-2012-10-461848 23343832 PMC3700465

[26] van de Donk NW Dhimolea E : Brentuximab vedotin. *MAbs.* 2012; 4(4): 458–65. 10.4161/mabs.20230 22684302 PMC3499340

[27] Sabattini E Pizzi M Tabanelli V : CD30 expression in peripheral T-cell lymphomas. *Haematologica.* 2013; 98(8): e81–2. 10.3324/haematol.2013.084913 23716537 PMC3729886

[28] Shustov A Cabrera ME Civallero M : ALK-negative anaplastic large cell lymphoma: features and outcomes of 235 patients from the International T-Cell Project. *Blood Adv.* 2021; 5(3): 640–648. 10.1182/bloodadvances.2020001581 33560375 PMC7876884

[29] Horwitz S O'Connor OA Pro B : The ECHELON-2 Trial: 5-year results of a randomized, phase III study of brentuximab vedotin with chemotherapy for CD30-positive peripheral T-cell lymphoma. *Ann Oncol.* 2022; 33(3): 288–298. 10.1016/j.annonc.2021.12.002 34921960 PMC9447792

[30] Cederleuf H Pedersen MB Jerkeman M : The addition of etoposide to CHOP is associated with improved outcome in ALK+ adult anaplastic large cell lymphoma: A Nordic Lymphoma Group study. *Br J Haematol.* 2017; 178(5): 739–746. 10.1111/bjh.14740 28485010

[31] Brink M Meeuwes FO van der Poel MWM : Impact of etoposide and ASCT on survival among patients aged <65 years with stage II to IV PTCL: a population-based cohort study. *Blood.* 2022; 140(9): 1009–1019. 10.1182/blood.2021015114 35544601 PMC9437712

[32] Deng S Lin S Shen J : Comparison of CHOP vs CHOPE for treatment of peripheral T-cell lymphoma: a meta-analysis. *Onco Targets Ther.* 2019; 12: 2335–2342. 10.2147/OTT.S189825 30992670 PMC6445243

[33] Abeyakoon C van der Weyden C Harrop S : Role of Haematopoietic Stem Cell Transplantation in Peripheral T-Cell Lymphoma. *Cancers (Basel).* 2020; 12(11): 3125. 10.3390/cancers12113125 33114606 PMC7692733

[34] Moskowitz CH Bertino JR Glassman JR : Ifosfamide, carboplatin, and etoposide: a highly effective cytoreduction and peripheral-blood progenitor-cell mobilization regimen for transplant-eligible patients with non-Hodgkin's lymphoma. *J Clin Oncol.* 1999; 17(12): 3776–85. 10.1200/JCO.1999.17.12.3776 10577849

[35] Moskowitz AJ Ghione P Jacobsen E : A phase 2 biomarker-driven study of ruxolitinib demonstrates effectiveness of JAK/STAT targeting in T-cell lymphomas. *Blood.* 2021; 138(26): 2828–2837. 10.1182/blood.2021013379 34653242 PMC8718625

[36] Smith SM Burns LJ van Besien K : Hematopoietic cell transplantation for systemic mature T-cell non-Hodgkin lymphoma. *J Clin Oncol.* 2013; 31(25): 3100–9. 10.1200/JCO.2012.46.0188 23897963 PMC3753702

[37] Furqan F Ahn KW Chen Y : Allogeneic haematopoietic cell transplant in patients with relapsed/refractory anaplastic large cell lymphoma. *Br J Haematol.* 2023; 200(1): 54–63. 10.1111/bjh.18467 36120837 PMC9772096

[38] Du J Yu D Han X : Comparison of Allogeneic Stem Cell Transplant and Autologous Stem Cell Transplant in Refractory or Relapsed Peripheral T-Cell Lymphoma: A Systematic Review and Meta-analysis. *JAMA Netw Open.* 2021; 4(5): e219807. 10.1001/jamanetworkopen.2021.9807 34042995 PMC8160596

[39] Zhang S Gu C Huang L : The third-generation anti-CD30 CAR T-cells specifically homing to the tumor and mediating powerful antitumor activity. *Sci Rep.* 2022; 12(1): 10488. 10.1038/s41598-022-14523-0 35729339 PMC9213494

[40] Merino M Kasamon Y Li H : FDA approval summary: Crizotinib for pediatric and young adult patients with relapsed or refractory systemic anaplastic large cell lymphoma. *Pediatr Blood Cancer.* 2022; 69(8): e29602. 10.1002/pbc.29602 35561013

[41] Redaelli S Farina F Stasia A : High Response Rates To Crizotinib In Advanced, Chemoresistant ALK+ Lymphoma Patients. *Blood.* 2013; 122(21): 368. 10.1182/blood.V122.21.368.368

[42] Rindone G Aroldi A Bossi E : A monocentric analysis of the long-term safety and efficacy of crizotinib in relapsed/refractory ALK^+^ lymphomas. *Blood Adv.* 2023; 7(3): 314–316. 10.1182/bloodadvances.2022007538 35914224 PMC9898594

[43] Richly H Kim TM Schuler M : Ceritinib in patients with advanced anaplastic lymphoma kinase-rearranged anaplastic large-cell lymphoma. *Blood.* 2015; 126(10): 1257–8. 10.1182/blood-2014-12-617779 26337354 PMC4559938

[44] Hida T Nokihara H Kondo M : Alectinib versus crizotinib in patients with *ALK*-positive non-small-cell lung cancer (J-ALEX): an open-label, randomised phase 3 trial. *Lancet.* 2017; 390(10089): 29–39. 10.1016/S0140-6736(17)30565-2 28501140

[45] Fukano R Mori T Sekimizu M : Alectinib for relapsed or refractory anaplastic lymphoma kinase-positive anaplastic large cell lymphoma: An open-label phase II trial. *Cancer Sci.* 2020; 111(12): 4540–4547. 10.1111/cas.14671 33010107 PMC7734006

[46] Knörr F Schellekens KPJ Schoot RA : Combination therapy with crizotinib and vinblastine for relapsed or refractory pediatric ALK-positive anaplastic large cell lymphoma. *Haematologica.* 2023; 108(5): 1442–1446. 10.3324/haematol.2022.281896 36519329 PMC10153539

[47] Lowe EJ Reilly AF Lim MS : Brentuximab vedotin in combination with chemotherapy for pediatric patients with ALK^+^ ALCL: results of COG trial ANHL12P1. *Blood.* 2021; 137(26): 3595–3603. 10.1182/blood.2020009806 33684925 PMC8462406

[48] Lowe EJ Reilly AF Lim MS : Crizotinib in Combination With Chemotherapy for Pediatric Patients With ALK+ Anaplastic Large-Cell Lymphoma: The Results of Children's Oncology Group Trial ANHL12P1. *J Clin Oncol.* 2023; 41(11): 2043–2053. 10.1200/JCO.22.00272 36534942 PMC10082271

[49] Damm-Welk C Kutscher N Zimmermann M : Quantification of minimal disseminated disease by quantitative polymerase chain reaction and digital polymerase chain reaction for *NPM-ALK* as a prognostic factor in children with anaplastic large cell lymphoma. *Haematologica.* 2020; 105(8): 2141–2149. 10.3324/haematol.2019.232314 31649129 PMC7395281

[50] Damm-Welk C Lovisa F Contarini G : Quantification of Minimal Disease by Digital PCR in ALK-Positive Anaplastic Large Cell Lymphoma: A Step towards Risk Stratification in International Trials? *Cancers (Basel).* 2022; 14(7): 1703. 10.3390/cancers14071703 35406475 PMC8996924

[51] Damm-Welk C Mussolin L Zimmermann M : Early assessment of minimal residual disease identifies patients at very high relapse risk in *NPM-ALK*-positive anaplastic large-cell lymphoma. *Blood.* 2014; 123(3): 334–7. 10.1182/blood-2013-09-526202 24297868

[52] Ait-Tahar K Damm-Welk C Burkhardt B : Correlation of the autoantibody response to the ALK oncoantigen in pediatric anaplastic lymphoma kinase-positive anaplastic large cell lymphoma with tumor dissemination and relapse risk. *Blood.* 2010; 115(16): 3314–9. 10.1182/blood-2009-11-251892 20185586

[53] Mussolin L Damm-Welk C Pillon M : Use of minimal disseminated disease and immunity to NPM-ALK antigen to stratify ALK-positive ALCL patients with different prognosis. *Leukemia.* 2013; 27(2): 416–22. 10.1038/leu.2012.205 22907048

[54] Sibon D Lamant L Vergé V : The ALK-OBS Trial: Results of a Multicenter Prospective Study Assessing the Prognostic Value of New Markers in Adults with ALK-Positive ALCL Treated By CHOEP: A Lysa Study. *Blood.* 2022; 140(Supplement 1): 3680–3682. 10.1182/blood-2022-170841

[55] Sibon D Fournier M Brière J : Long-term outcome of adults with systemic anaplastic large-cell lymphoma treated within the Groupe d'Etude des Lymphomes de l'Adulte trials. *J Clin Oncol.* 2012; 30(32): 3939–46. 10.1200/JCO.2012.42.2345 23045585

[56] Morel A Brière J Lamant L : Long-term outcomes of adults with first-relapsed/refractory systemic anaplastic large-cell lymphoma in the pre-brentuximab vedotin era: A LYSA/SFGM-TC study. *Eur J Cancer.* 2017; 83: 146–153. 10.1016/j.ejca.2017.06.026 28735072

[57] Pro B Advani R Brice P : Five-year results of brentuximab vedotin in patients with relapsed or refractory systemic anaplastic large cell lymphoma. *Blood.* 2017; 130(25): 2709–2717. 10.1182/blood-2017-05-780049 28974506 PMC5746164

